# Data in Personalized Nutrition: Bridging Biomedical, Psycho-behavioral, and Food Environment Approaches for Population-wide Impact

**DOI:** 10.1016/j.advnut.2025.100377

**Published:** 2025-01-20

**Authors:** Jakob Linseisen, Britta Renner, Kurt Gedrich, Jan Wirsam, Christina Holzapfel, Stefan Lorkowski, Bernhard Watzl, Hannelore Daniel, Michael Leitzmann, Working Group “Personalized Nutrition” of the German Nutrition Society

**Affiliations:** 1 Epidemiology, Medical Faculty, University of Augsburg, University Hospital Augsburg, Augsburg, Germany; 2 Institute of Information Processing, Biometry and Epidemiology, Ludwig-Maximilians University, Munich, Germany; 3 Department of Psychology, University of Konstanz, Konstanz, Germany; 4 Centre for the Advanced Study of Collective Behaviour, University of Konstanz, Konstanz, Germany; 5 Technical University of Munich, ZIEL – Institute for Food & Health, Research Group Public Health Nutrition, Freising, Germany; 6 Operations and Innovation Management, HTW Berlin, Berlin, Germany; 7 Institute for Nutritional Medicine, Technical University of Munich, School of Medicine and Health, Munich, Germany; 8 Department of Nutritional, Food and Consumer Sciences, Fulda University of Applied Sciences, Fulda, Germany; 9 Institute of Nutritional Sciences, Friedrich Schiller University, Jena, Germany; 10 Department of Physiology and Biochemistry of Nutrition, Max Rubner-Institut, Karlsruhe, Germany; 11 Technical University of Munich, Freising, Germany; 12 Department of Epidemiology and Preventive Medicine, University of Regensburg, Regensburg, Germany

**Keywords:** personalized nutrition, precision nutrition, biomedical, environmental data, behavior change, food environment, dynamic system, advice, digital ecosystem, *A*PNAS

## Abstract

Personalized nutrition (PN) represents an approach aimed at delivering tailored dietary recommendations, products, or services to support both prevention and treatment of nutrition-related conditions and to improve individual health using genetic, phenotypic, medical, nutritional, and other pertinent information. However, current approaches have yielded limited scientific success in improving diets or in mitigating diet-related conditions. In addition, PN currently caters to a specific subgroup of the population rather than having a widespread impact on diet and health at a population level. Addressing these challenges requires integrating traditional biomedical and dietary assessment methods with psycho-behavioral, and novel digital and diagnostic methods for comprehensive data collection, which holds considerable promise in alleviating present PN shortcomings. This comprehensive approach not only allows for deriving personalized goals (“what should be achieved”) but also customizing behavioral change processes (“how to bring about change”). We herein outline and discuss the concept of “Adaptive Personalized Nutrition Advice Systems,” which blends data from 3 assessment domains: *1*) biomedical/health phenotyping; *2*) stable and dynamic behavioral signatures; and *3*) food environment data. Personalized goals and behavior change processes are envisaged to no longer be based solely on static data but will adapt dynamically in-time and in-situ based on individual-specific data. To successfully integrate biomedical, behavioral, and environmental data for personalized dietary guidance, advanced digital tools (e.g., sensors) and artificial intelligence-based methods will be essential. In conclusion, the integration of both established and novel static and dynamic assessment paradigms holds great potential for transitioning PN from its current focus on elite nutrition to a widely accessible tool that delivers meaningful health benefits to the general population.

**Statements of significance**

This perspective proposes a comprehensive framework for personalized nutrition (PN) that integrates biomedical, psycho-behavioral, and environmental data using advanced digital and artificial intelligence-based tools, with the potential to expand PN’s impact from niche applications to population-wide health benefits.

## Introduction

Personalized nutrition (PN), now more frequently referred to as precision nutrition (PrN), aims to tailor dietary advice or products to individuals’ specific needs, goals, and expectations. Thus far, PN concepts have primarily focused on genetic variants and/or the gut microbiome, often including only a limited range of additional information, such as anthropometric measures or dietary intake [1]. PrN has taken a step further in this direction by incorporating more comprehensive phenotype data and integrating findings from omics technologies, such as epigenetics, proteomics, and metabolomics [2].

Although the allure of tailoring a diet to an individual’s unique genetic and metabolic profile holds promise for improving current health status, the scientific validation supporting these claims is often lacking, and available studies are inconclusive [3]. Few scientific projects have tested the feasibility and efficacy of PN programs. The largest investigation of PN to date is the Food4Me study, a pan-European endeavor carried out under the auspices of an EU framework. The principal finding of this study was that PN, in itself, led to improved diet and health indicators. However, the inclusion of sophisticated parameters such as blood parameters or gene variants did not significantly improve dietary behavior [4]. This conclusion is in line with findings from recent systematic reviews of human intervention studies, which reported disappointing results regarding the efficacy of PN protocols [5,6]. These setbacks warrant the exploration of novel avenues in PN, particularly when one goal is to enhance public health.

Although the effectiveness of PN in promoting a sustained change in dietary behavior or lifestyle has not yet been proven through well-designed intervention studies, there is great public interest in a more personalized diet [7]. The reasons why people are interested in or seek PN advice or products vary. Personal motivation for PN can result from specific disease and health issues, excess body weight, or physical and cognitive performance limitations [8]. Moreover, the desire to improve one’s own lifestyle, overall health, and wellbeing is also an important factor [9]. This indicates a general need for more specific information about the healthiness of one's diet and a belief that dietary changes are necessary to achieve better or optimal health benefits. Despite these varied reasons for interest in PN advice and products, PN clients often belong to higher education and income groups [10]. Most commercial offerings in the PN sector are expensive for clients and are rarely reimbursed by health insurance companies. Consequently, PN currently caters to a specific subgroup of the population rather than having a broader impact on diet and health at the population level.

**FIGURE 1:**
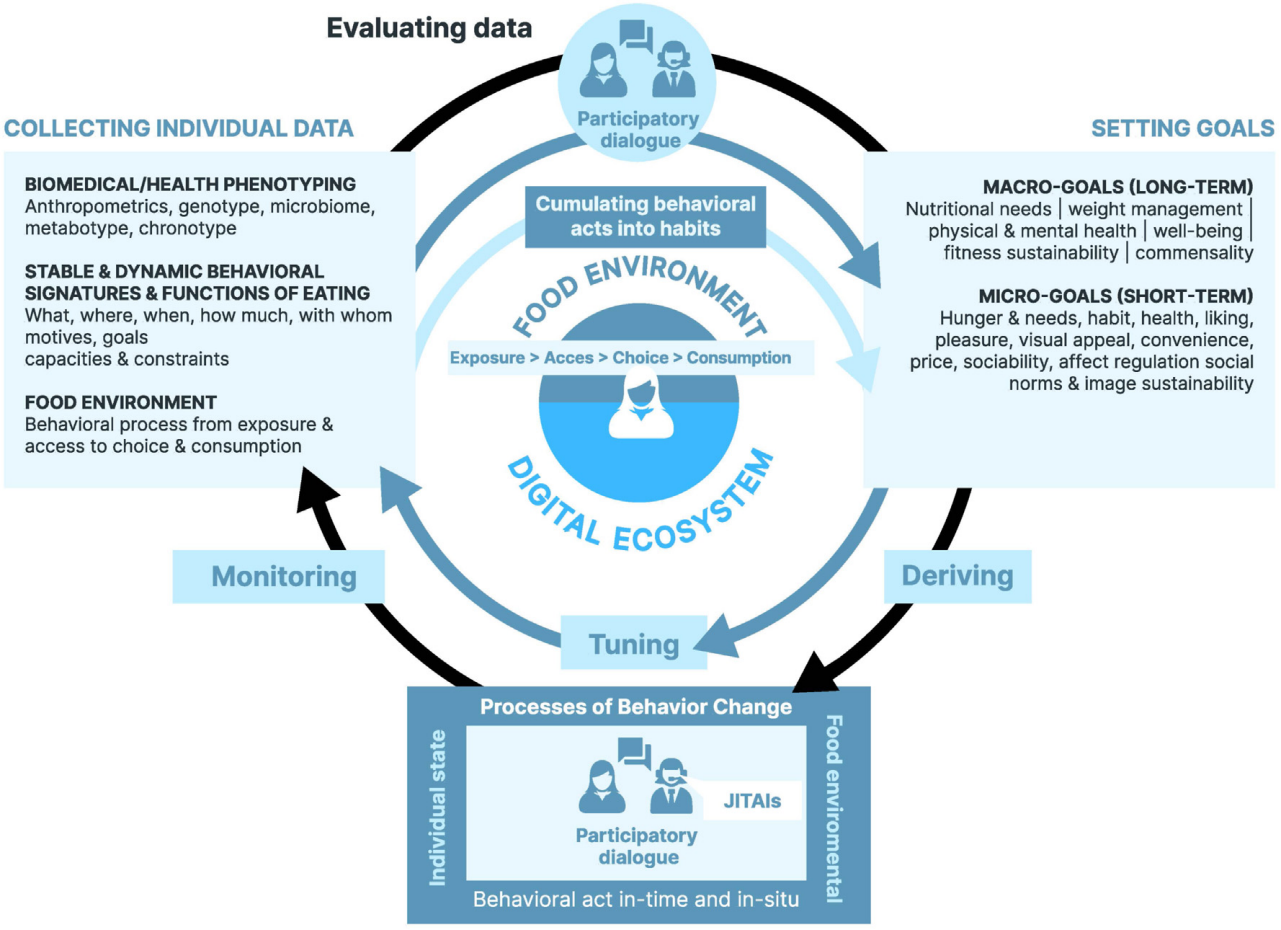
Framework of the “Adaptive Personalized Nutrition Advice Systems” (*A*PNAS) (© 2023 Renner et al., 2023. Published by Elsevier Inc. on behalf of American Society for Nutrition).

In view of the limited success and reach of current PN approaches, a novel framework called Adaptive Personalized Nutrition Advice Systems (*A*PNASs) has been proposed (Figure 1) [11]. Extending beyond current approaches to PN, which focus on refining individual biomedical-based diet goals through multi-omics profiling, *A*PNASs also aim at personalizing how consumers and patients apply the given advice in their daily lives. *A*PNASs suggest that the personalization of nutrition advice should relate not only to deriving personalized goals (“what to achieve”) but also to personalizing the process of behavioral change (“how to change”) (see also [9]). Accordingly, this approach places people at the center, considering their abilities, capacities, goals, and constraints within their daily lives and social contexts. Specifically, *A*PNASs’ focus on setting personalized goals and tailoring adaptive processes of behavior change. Notably, depending on the individual goals and preferences, *A*PNASs may even utilize minimal genotype and omics-based data, making a shift from a predominantly biomedical to a more intensive behavioral framework for PN. Therefore, in addition to collecting individual data for in-depth genetic and metabolic phenotyping, as suggested by current PN approaches, *A*PNASs emphasize in-depth profiling of individual behavioral signatures and food environments [11]. This approach raises the question of what types of data could be most effectively utilized for PN.

Using *A*PNASs as a framework, this work aims to *1*) outline the different types of data entailed in PN, ranging from biomedical and behavioral to food environment data, across various spatial and temporal scales, and *2*) explore the current and future possibilities offered by digital and analytical tools for a more widespread impact of PN on the population level.

### Types of data

*A*PNASs identify 3 distinct assessment domains, each encompassing different types of data (Figure 1): *1*) biomedical/ health phenotyping, *2*) stable and dynamic behavioral signatures, including functions of eating, and *3*) the food environment.

**FIGURE 2:**
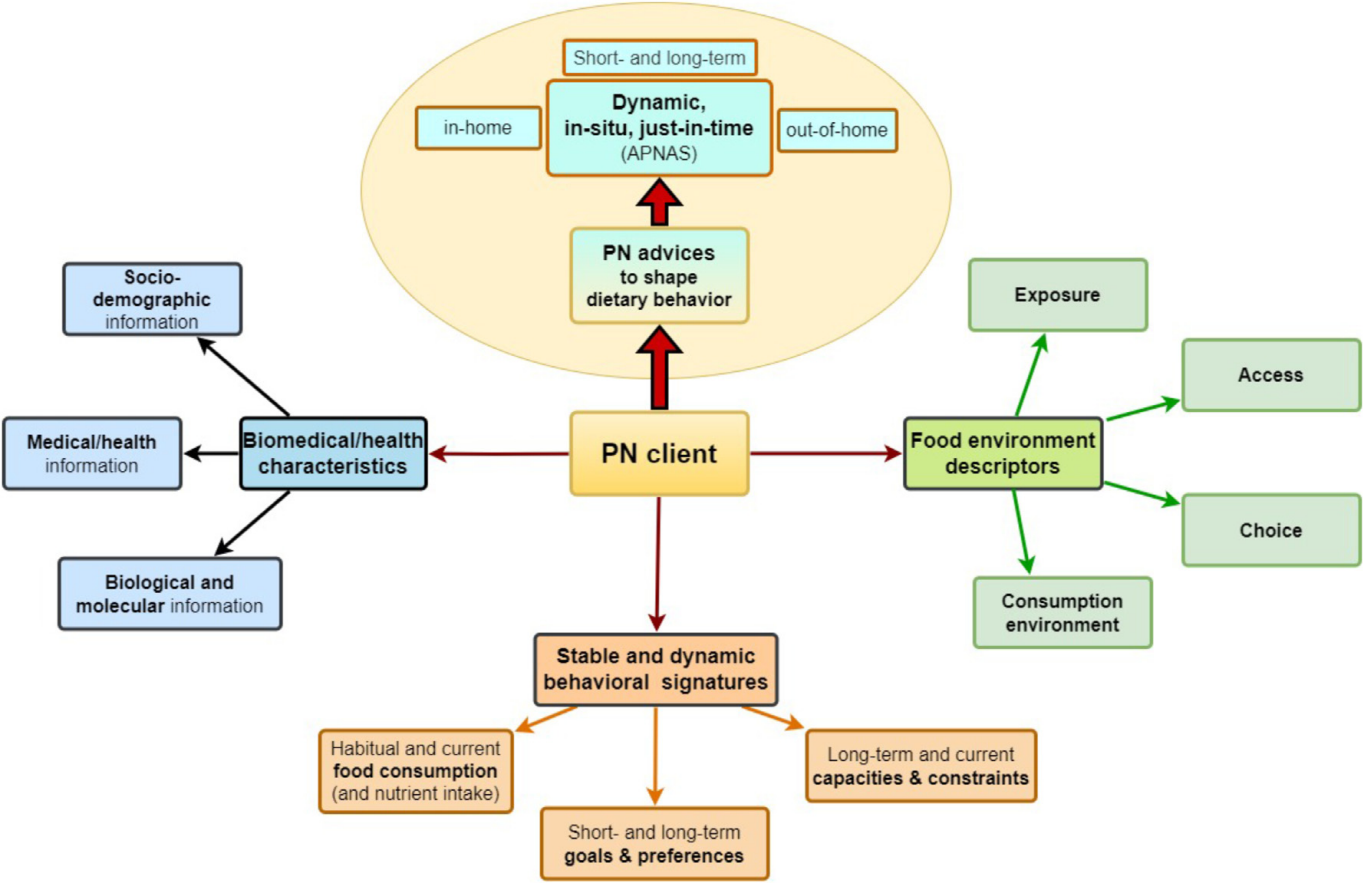
Overview of data assessment domains to derive static and dynamic personalized nutrition (PN) advice to shape a person’s dietary behavior.

As an initial step, biomedical and health phenotyping is conducted, along with profiling of individual behavioral signatures and the food environment. This begins with relatively stable personal characteristics and food environment factors to derive individual goal preferences and identify initial leverage points for behavioral change processes (see also approaches to solve the “cold start” problem in computer-based information systems, such as digital recommender systems [Computer-based information systems, involving a degree of automated data modelling, can only make inferences for applications or users based on the information available. The ‘cold start’ problem refers to the challenge these systems face in making personalized inferences for users when they have not yet accumulated sufficient data.]). This step is dynamically enhanced by the collection of real-time, context-specific individual data, which personalizes goals and refines just-in-time adaptive interventions (JITAIs; see [12]) to better support behavioral change. Thus, data collection for personalizing goals and behavior change processes is envisaged to be dynamic and adaptive, not just stable or static. This involves collecting data in real-time (in-time) and in the relevant context (in-situ), with the frequency and timing tailored to individual needs and preferences, enabling goals to be updated dynamically based on real-time inputs. Recent technological advancements have made it possible to gather an unprecedented amount of both static and dynamic behavioral and health data in this manner (Figure 2). Although there is interest in PN approaches and a willingness to provide personal data, the extent to which individuals are prepared to share their data for tailored PN advice or products is not entirely clear. Factors such as the perceived benefits of PN, trust in the organization collecting the data, and assurances about data security and ethical use are critical in influencing this decision-making process. Privacy protection concerns, including the potential misuse of data, unauthorized access, and lack of transparency about data handling, also play a significant role [13].

### Assessment domain “biomedical/health characteristics”

Similar to diagnostic processes in various biomedical and health domains (e.g., Dietetic Care Process [14,15]), the initial stage of the *A*PNASs entails the assessment of data, including *1*) sociodemographic and basic data, *2*) the current medical/health status, as well as *3*) current biological and molecular data.

In the following, we describe these types of data and their significance in the context of PN (see also Table 1). Although certain parameters are static and remain (relatively) constant, requiring measurement only once (e.g., sex, genotypic information, chronotype), others are more dynamic and necessitate repeated or continuous assessments, such as metabolites or biomarkers. Moreover, depending on the health situation of participants, certain exclusion criteria may need to be applied to prevent legal or ethical complications arising from PN advice, products, or services [16]. These include but are not limited to eating disorders, medication interactions, and severe mental health conditions. Clearly outlining these criteria upfront is advisable. Additionally, involving medical experts is recommended for addressing these and other aspects of the proposed concept. Notably, mental health issues such as depression, social anxiety, and attention-deficit/hyperactivity disorders are often more prevalent among individuals with eating disorders, complicating the safe implementation of PN strategies in these cases [17].

**TABLE 1:** Assessment domain “biomedical/health characteristics”.

Type of data					
Sociodemographic and basic	Medical/health	Biomedical and molecular			
		Anthropometry	Clinical laboratory analyses	Omics analyses	(Continuous) monitoring of nutritional status
Gender	Individual and family history of diseases	Body weight and height	Biomarkers of nutrient status	Genome	Bodily metabolites
Age	Food allergies/intolerances	Body fat mass (total, regional)	Clinical biochemistry	Gut microbiome	Bodily functions
Education	Rare diet-related diseases (e.g., PKU)	Waist circumference		Epigenome	
Language and communication skills	Metabolic diseases	Muscle mass		Transcrip-tome	(Physical activity)
	Other major diseases			Proteome	
(Household) Income	Current medication			Metabolome	
Employment	Physical disability, immobility				
Occupation	Pregnancy, lactation				

#### Sociodemographic data

A primary goal of a healthy diet is to fulfill essential nutrient requirements to prevent deficiencies and reduce the risk of diseases. Dietary reference values for energy and nutrient intake are provided separately for men and women, different age groups, and individuals in specific situations (e.g., pregnant or breastfeeding women) [18,19]. Thus, information on (stable) individual characteristics, such as sex and age, is essential for PN considerations. These reference values are designed for healthy individuals in the population. The associated recommended dietary allowances include a safety margin (e.g., ideally average requirement plus 2SDs) to ensure that nearly all individuals within different population subgroups meet their specific needs [20].

Education, language and communication skills, and literacy play a critical role in processing, understanding, and utilizing the information, products, or services offered as part of PN. Communication skills are crucial for effectively expressing and exchanging information, which is important for a positive and effective advisor-advisee or patient-doctor relationship. Literacy, however, is predominantly about understanding and using (health) information. Currently, different scopes of literacy, such as health, food, nutrition, and media literacy, are being discussed. These emphasize distinct types of knowledge essential for promoting health-related outcomes [21]. Especially noteworthy is that food literacy [22] can significantly influence the effectiveness of PN.

In addition, cultural norms and traditions shape food choices, meal patterns, and attitudes toward dietary changes. Traditional foods, religious practices, and communal habits influence what is acceptable within specific contexts [23]. Understanding these factors is crucial for practical and respectful PN strategies. Additionally, agency—the ability to make independent choices—moderates behavior change, with resources, autonomy, and social support playing key roles in implementing dietary changes [24].

Individual income and wealth can significantly influence an individual’s access to PN services. Financial stress, indicative of the balance between income and necessary expenses, is a key factor. This is often reflected by the available budget at the end of each month. These variables are frequently assessed under the umbrella term “socioeconomic status,” which is defined by household income, education, and occupation [25]. However, amalgamating these variables may confound the distinct ways in which education and income-related individual characteristics affect an individual’s access to PN services.

#### Medical/health status data

The assessment of health status, encompassing medical conditions, family history of diseases, allergies, and any medical support received, is crucial owing to its potential impact on dietary and lifestyle guidance. Constructing dietary advice also requires basic information, such as details about physical disabilities and the current physiological status (e.g., pregnancy).

Diseases influenced by dietary factors are particularly relevant for PN. Key details include allergies and intolerances to specific foods or food components, information essential for dieticians, and PN professionals (Table 1) [26]. Among the most common noncommunicable diseases linked to diet are metabolic conditions including obesity, type 2 diabetes mellitus, hyperuricemia and gout, dyslipidemia, and hypertension. In addition, knowledge about rare metabolic disorders requiring strict dietary adherence, such as phenylketonuria, is indispensable.

#### Biological and molecular data

Obesity, especially the accumulation of excess visceral body fat, demands particular attention in PN guidance, as it is a major factor impairing health [27]. Although obesity prevalence and severity vary across population groups, surrogates for central adiposity, such as waist circumference, waist-to-hip ratio, and height-to-waist ratio, are valuable tools that provide critical insights into abdominal fat distribution not captured by BMI. To gather precise data, employing technician-assessed anthropometry measurements is preferred over relying on self-reported estimates and simple calculations the of BMI.

Furthermore, clinical biochemistry data add valuable information, including circulating levels of lipids and lipid fractions, fasting or random plasma glucose, Glycated haemoglobin, uric acid, and markers of liver and kidney function. Mobile sensors and wearable devices with high temporal-resolution tracking of multiple health parameters, including readings like pulse rate, blood oxygen levels, glucose concentrations, and electrocardiograms, offer dynamic and continuous insights into an individual's health status [[28], [29], [30]].

A new foundation of PN is advanced genetic and metabolic phenotyping, often encompassed under the terms “omics data” or “multi-omics data.” Although these terms lack a precise scientific definition, they refer to high-throughput and high-density analyses of entities that represent the genome in its expression at the levels of proteins and metabolites. This includes factors like epigenetic marks, parts or the entirety of the transcriptome, the proteome, and the pool of metabolites. Modern applications also incorporate the microbiome at the genetic and predicted functional levels [31]. Studies have successfully demonstrated the capability of phenotyping an individual using such detailed read-outs [32]. However, despite these advancements, achieving a rapid and thorough understanding of how these genetic and metabolic signatures correlate with health or disease trajectories remains challenging. The field of “multi-omics” still represents a costly endeavor, fraught with numerous complexities and limitations, including challenges related to reproducibility [33]. The unique attributes and constraints of each multi-omics technique necessitate the use of artificial intelligence (AI) tools for data aggregation, analysis, and interpretation [34]. Of note, integrating expansive omics-based datasets into the context of PN is yet to be realized.

Well-established markers that reflect nutrient status are not covered by omics platforms; this is a critical shortcoming and applies to the majority of vitamins, minerals, and trace elements. Moreover, current metabolite profiling lacks precise determination of actual concentrations, crucial for clinical diagnostics. Similarly, microbiome signatures derived from stool samples typically provide information on relative abundance, rather than absolute densities of bacteria [35]. Nevertheless, the prospect of more sophisticated phenotyping methods and more valid biomarkers offers a novel source of higher-quality data, enabling more accurate classification of individuals for personalized strategies [33,34].

Metabolite profiling augments conventional food intake assessments by analyzing food-specific exposure markers found in plasma and/or urine. These biomarkers reveal recent food or beverage consumption and offer a valuable perspective on dietary behavior [[36], [37], [38]].

In addition, the concept of metabotypes*,* which integrates blood and urine metabolite profiling with clinical parameters such as blood glucose and cholesterol, enables the identification of metabolically similar groups of people [39,40]. Such information can feed risk scores to classify people according to their risk of developing noncommunicable diseases such as type 2 diabetes mellitus or cardiovascular disease. Moreover, this approach can identify specific subgroups that stand to benefit the most from targeted dietary interventions [41,42].

Incorporating biomarkers of essential nutrients, such as vitamins, minerals, and trace elements, is often overlooked in current phenotyping applications. For these nutrients, distinct technologies, such as inductively coupled plasma mass spectrometry, are required to obtain data on multiple elements from a single sample [43]. Although only a few providers of PN services presently integrate such data, their inclusion could provide valuable insights. However, collecting and analyzing biomaterials, especially blood, entail challenges despite available innovative techniques like dried blood spots or sponges for minimally invasive blood collection.

In addition, these laboratory analyses often limit PN accessibility to consumers because of their cost. Expanding the reach of PN may demand more affordable technologies, like sensors based on molecular electronics ([44], see also [34]). These sensors hold potential, albeit still in an early developmental stage.

### Assessment domain “sable and dynamic behavioral signatures”

Under the *A*PNASs framework [11], the initial stage involves profiling of *1*) individual behavioral habits and signatures, along with determinants of behavior such as *2*) goals and preferences, and *3*) capacities and constraints. These serve as leverage points for initiating processes of behavioral change (Table 2). Although some aspects of these 3 factors remain relatively stable over time and across various circumstances (e.g., food restrictions, predisposition for stress-eating), providing critical initial entrance points for initiating processes of behavioral change, other factors are dynamic and necessitate repeated or continuous assessments, allowing JITAIs to increasingly adapt the behavioral change processes to the individual (see also [45,46]).

**TABLE 2:** Assessment domain “stable and dynamic dietary behavioral signatures”.

Short- and long-term individual behaviors and signatures		Goals and preferences	Capacities and constraints
Food consumption	Meal characteristics		
Habitual food consumption, nutrient intake, dietary patterns	Habitual meal timing, meal sequence, meal composition	Specific type of diet (vegetarian, vegan, religion, ethnicity)	Food literacy, cooking skills
Current food consumption, nutrient intake	Actual meal timing, meal situation, meal composition	Food acceptance and preferences	Use of delivery services, out-of-home consumption
Biomarkers of food or nutrient intake (e.g., glucose monitoring)	Type and frequency of snacking	Long-term goals (macro-goals): health-related (e.g., body weight change, fitness, wellbeing); sustainability and lifestyle related (e.g., reducing carbon foot print, better animal welfare)	Financial situation, circadian rhythm, sleep
		Short-term goals (micro-goals): eating motives in-the-moment (e.g., liking, convenience, affect regulation, price, sociability)	

#### Individual behavioral signatures and habits

Collecting information on dietary habits is fundamental for effective professional dietary counseling. In PN, baseline information gathering includes identifying food items or food groups that are restricted because of cultural factors, social norms, personal values, and beliefs (e.g., kosher diets, veganism).

Evaluating meal and snack composition might involve listing consumed food items without specifying precise quantities [47]. This can also include information on food preferences, as well as meal timing and sequence throughout the day [48]. In addition, information about the frequency and location of eating out-of-home or using food delivery services has become an important aspect of daily food consumption. Such data may be self-reported or may be obtained from service providers (Table 2). Service providers, such as restaurants, food delivery platforms, or catering companies, may provide information on order details and consumption patterns from their databases upon authorized request.

For assessing habitual food consumption and estimating nutrient intake, standard methods involve food-frequency questionnaires. Current eating patterns are typically captured using repeated 24-h dietary recalls and records of estimated or weighed food consumption over several days (selected randomly over a defined period) [49]. Precise recording of actual food consumption is also possible. Traditional paper-based questionnaires are increasingly being replaced by digital solutions, such as smartphone apps or web-based tools [[50], [51], [52], [53]]. These digital methods offer enhanced convenience and functionality but still come with certain limitations, including recall bias, underreporting, and portion size inaccuracies, which require a scientific evaluation of their relative validity and reproducibility.

Among these digital advancements, data generated through the use of digital food images has gained significant attention for its potential to improve the precision and accuracy of dietary assessments. This method can assist, either actively or passively (with or without user input), in estimating intake and portion sizes, thereby enhancing the precision of dietary reporting. Image-based food recognition, volume estimations, and subsequent nutrient and energy intake assessments are increasingly automated through computer vision-based applications [52,54]. These applications leverage AI, utilizing machine learning techniques, including deep learning (DL), to recognize food items and estimate volume to predict the nutritional value of a depicted meal or food item [54]. However, AI systems, although promising, depend on user input and face challenges like food recognition errors, lack of standardization, and “black box” decision-making, as the underlying factors driving the algorithm’s decision-making process remain unclear. Amugongo et al. [55] argue that AI-powered systems should provide explanations for their classifications or estimations to enhance transparency for users. The pursuit of increased transparency and interpretability lies at the core of explainable AI, which is crucial for improving the trustworthiness of AI systems. Despite their inherent limitations, these techniques provide a vast amount of different types of data, thereby offering new and valuable insights into food choices, dietary patterns, and potential health risks. AI-based solutions will increasingly facilitate rapid aggregation and evaluation of such data [56]. Over time, self-learning AI systems can construct an exhaustive profile of an individual’s dietary habits and variability of daily eating behavior, adapting based on the evolving information provided.

People’s decisions about eating extend beyond just what and how much they eat; they also encompass where, when, how, and with whom they eat or do not eat, constituting idiosyncratic behavioral signatures [11]. High-resolution behavior assessments conducted in-situ and in-time in natural settings, utilizing mobile sensors, can capture these individual behavioral signatures. For example, employing ecological momentary assessment (EMA) contingent on eating events has revealed considerable inter- and intraindividual differences in eating behavior over time [57,58]. Hence, eating behavior is highly dynamic because it varies not only between but also within individuals. For effective long-term behavior change, it is important to enable individuals to act in-the-moment and in-situ (“behavioral act”) and to cumulate behavioral acts into habitual, long-term behavioral patterns. This “small-changes” approach has gained considerable traction in numerous government and nongovernment initiatives [59]. Addressing elements of individual behavioral signatures (e.g., timing or duration of meals; skipping of meals) opens new avenues for personalized interventions aimed at behavior change. Although EMA captures valuable data, it may introduce reactivity bias and face technical issues like sensor malfunctions or inconsistent engagement, and its long-term success warrants confirmation.

#### Related behaviors

The most important determinant of differences in total energy requirements within specific sex and age groups is physical activity level. A lack of physical activity and prevalent sedentary behavior are recognized as risk factors for obesity and numerous chronic diseases. Thus, gathering information on an individual’s level of physical activity or inactivity, encompassing both long-term habits and current behaviors, is imperative. Validated questionnaires serve as a viable tool to assess habitual physical activity during work and leisure time across extended periods [60]. Numerous wearable devices are now available, furnished with features that enable continuous monitoring of various dimensions of physical activity [61]. However, physical activity questionnaires are prone to measurement errors, whereas wearable devices face challenges such as improper usage, calibration issues, and limited battery life, which can impact data quality. Beyond physical activity, other lifestyle factors also play a crucial role in health and nutrition. For instance, smoking is a significant health risk factor that affects nutrient levels, such as vitamin C status, information on smoking and smoking intensity is pertinent. Additional individual characteristics that could influence dietary behavior and metabolic health include circadian rhythm, and sleep duration and quality [62]. Consumer sleep-tracking devices are evolving rapidly, with some already demonstrating high accuracy in detecting sleep and wake phases [63,64].

Integrating dietary assessment and digital food images with other health data enables the identification of dietary components relevant to conditions such as diabetes or allergies, ensuring that dietary advice aligns with medical needs through integration with patient health records. These tools can also link nutrient intake with biomarkers like blood glucose or lipid levels, whereas combining microbiome data with meal composition provides insights into the diet’s impact on gut health. Additionally, digital food tracking can be combined with behavioral data, such as EMA, to identify patterns like stress-eating or irregular meal timing. Dietary data can also be merged with physical activity, sleep, and smoking data to generate a comprehensive view of health behaviors, enabling PN strategies to address multiple lifestyle factors simultaneously.

#### Goals and preferences

For PN to be effective, it must align with an individual’s needs, goals, and expectations. Eating behavior is determined by a multitude of factors [65,66]. Hence, in addition to primary motives such as hunger and taste, there are various other compelling reasons that determine what, how much, and how individuals eat. Studies have consistently identified 15 different eating motives or functions of normal eating (see also micro-goals in Figure 1) [[66], [67], [68]]. These eating motives include social reasons such as commensality, as well as environmental and sustainability concerns, which shape individual food choices. To develop effective PN solutions, it is crucial from an *A*PNASs perspective to incorporate individual goal preferences, including pleasure, commensality, and, most importantly, making sustainable dietary choices, alongside typical biomedical targets.

Moreover, individual goal preferences encompass long-term goals (macro-goals) like mental health, wellbeing, fitness, or enjoyment, as well as eating motives in-the-moment (micro-goals). These goals can vary significantly because of individual states and environments, necessitating dynamic adjustments to align macro- and micro-goals, reduce conflicts, and create synergies. Thus, the selection and prioritization of macro- and micro-goals should be tailored to an individual’s preference structure and capacities (see also Figure 1).

In a similar vein, some individuals may seek general advice focused on personal health or fitness, whereas others may prioritize hedonic or sustainability aspects. Next, some may require specific guidance, like selecting items in a supermarket or choosing meals at a restaurant. Therefore, PN must be designed to cater specifically to an individual’s goals, needs, and capacities. If not appropriately tailored, PN efforts risk causing confusion because of information overload or frustration stemming from insufficient information [69].

#### Capacities and constraints

Achieving sustainable behavioral change is inherently challenging, because it involves overcoming deeply ingrained habits and external barriers. For the personalization of behavioral change processes, it is essential to provide in-situ and just-in-time information in real-life food environments, addressing the “how” and “when” to change. This requires consideration of individual capacities and constraints, often referred to as “barriers and enablers” in the literature, across various contexts, such as self-regulation capacities, available behavioral options, and economic resources. Unlike generic approaches, behavior change strategies should be personalized by aligning them with these individual factors. For example, enhancing self-regulation capacity in stress-hyperphagic individuals in diverse contexts is crucial. In the realm of PN, Dijksterhuis et al. [8] have identified 4 psychosocial types of consumers, namely “intrinsic interest and capabilities for healthy eating,” “perceived difficulty to eat healthily,” “self-worth insecurity,” and “seeking positive challenges.” These types differ substantially in their preferences and needs of advice.

### Assessment domain “food environment”

The food environment, forming the backdrop of nutritional behavior (e.g., [70]), exerts a powerful influence on food choices and eating behaviors. In general, the food environment entails all environmental factors that impact nutritional behavior (Table 3). Consequently, eating results not only from decisions made at the moment of concrete consumption, but also from a behavioral process spanning 4 core phases: *1*) exposure (i.e., what people see and perceive in their daily environment shapes social norms); *2*) access (i.e., which foods are physically accessible and socially acceptable); *3*) choice (i.e., which products are selected or purchased); and *4*) consumption (i.e., which foods, meals, or snacks are actually eaten). For example, frequent exposure to fast-food outlets is associated with unhealthy diets and high rates of obesity (for a review, see [71]). Similarly, the social environment exerts a pervasive and powerful influence on what and how much people eat (for an overview, see [72]). For example, mealtimes, established as social norms, shape collective eating behaviors and social lives [73]. Therefore, integrating the environmental context into PN advice (i.e., where and when to eat) is a promising approach. Initial evidence for this concept comes from a recent study showing higher acceptance of PN advice at lunch compared to breakfast or dinner [74].

**TABLE 3:** Assessment domain “food environment”.

Exposure	Access	Choice	Consumption environment
Nearby shops (reachable by foot)	Costs of products	Sources of information	Ambience (e.g., noise level, smell. lighting)
Supermarkets, retailers, etc. (reachable by public transportation, car, bike)	Transportation costs	Social network, social acceptance	Time allocation
Eligibility to visit canteens	Available household budget	Companionship (family, friends, colleagues, etc.)	Plate size, portion size
Out-of-home consumption (fast-food restaurants, to-go stores, etc.)	Usage of digital devices and payment options	Cooking knowledge and preparedness	Social setting, e.g., dining companions
Use of delivery services		Preferences and requests within the household	

(Digital) data are provided by the individual itself but also via market partners (shops, restaurants, etc.), and by analysis of the food environment landscape.

The concept of guiding and supporting individuals throughout the entire behavioral process and consumption journey, from exposure and access to purchasing food, to meal preparation and consumption, aligns with and extends traditional dietary counseling practices. Hence, gathering information on the food environment is crucial, particularly in light of the increasing prevalence of home delivery services, ready-to-eat meals, and out-of-home consumption, which not only shape an individuals’ dietary patterns but also leaves data traces useful for PN [75].

Importantly, the information required for effective PN advice varies across different domains of the food environment. For example, in retail settings, factors such as price, location, availability, and the specific food choices made by consumers are highly relevant. Seasonal variations and cultural traditions (e.g., Thanksgiving, Christmas, Diwali) also play a significant role in influencing food availability and consumer behavior. Individual ordering data, often retained for financial records (e.g., delivery services, company or school cafeterias), can potentially be harnessed to feed future PN algorithms. Also, Global Positioning System (GPS) tracking can pinpoint food consumption locations and provide relevant data (as described above) to estimate meal quality and quantity [76]. These examples highlight the extensive data requirements and the need for ongoing utilization of technological devices to gather information and offer tailored guidance. The application of AI-based methods is essential to aggregate and integrate behavioral data, identify primary targets, and deliver suitable advice or products. Incorporating positive feedback that reflects progress toward established goals is advisable.

### Transitioning from static to a more dynamic PN: a future perspective

A starting point and minimum gold standard assessment for PN involves assessing static or relatively stable individual characteristics such as sex, age, BMI, waist circumference, physical activity, dietary preferences, and health limitations, including food allergies and intolerances. Incorporating information about habitual food preferences and goals is important to enhance acceptance and adherence to PN advice. Of note, disregarding these essential static data in recommendations could lead not just to limited effectiveness but also to potential legal repercussions for the advisor, such as liability if harm occurs because of ignored allergies or health limitations.

Implementing PN effectively, however, requires aligning shared goals between the advisor and the individual seeking counseling, a challenging task [77,78]. The process of defining goals requires the definition of an overarching macro-goal (e.g., body weight reduction), followed by realistic short- and medium-term aims (micro-goals). This process likely requires discussion between both counseling partners; it is the basis for evaluating the effectiveness of the PN for both the client and the PN provider.

**FIGURE 3:**
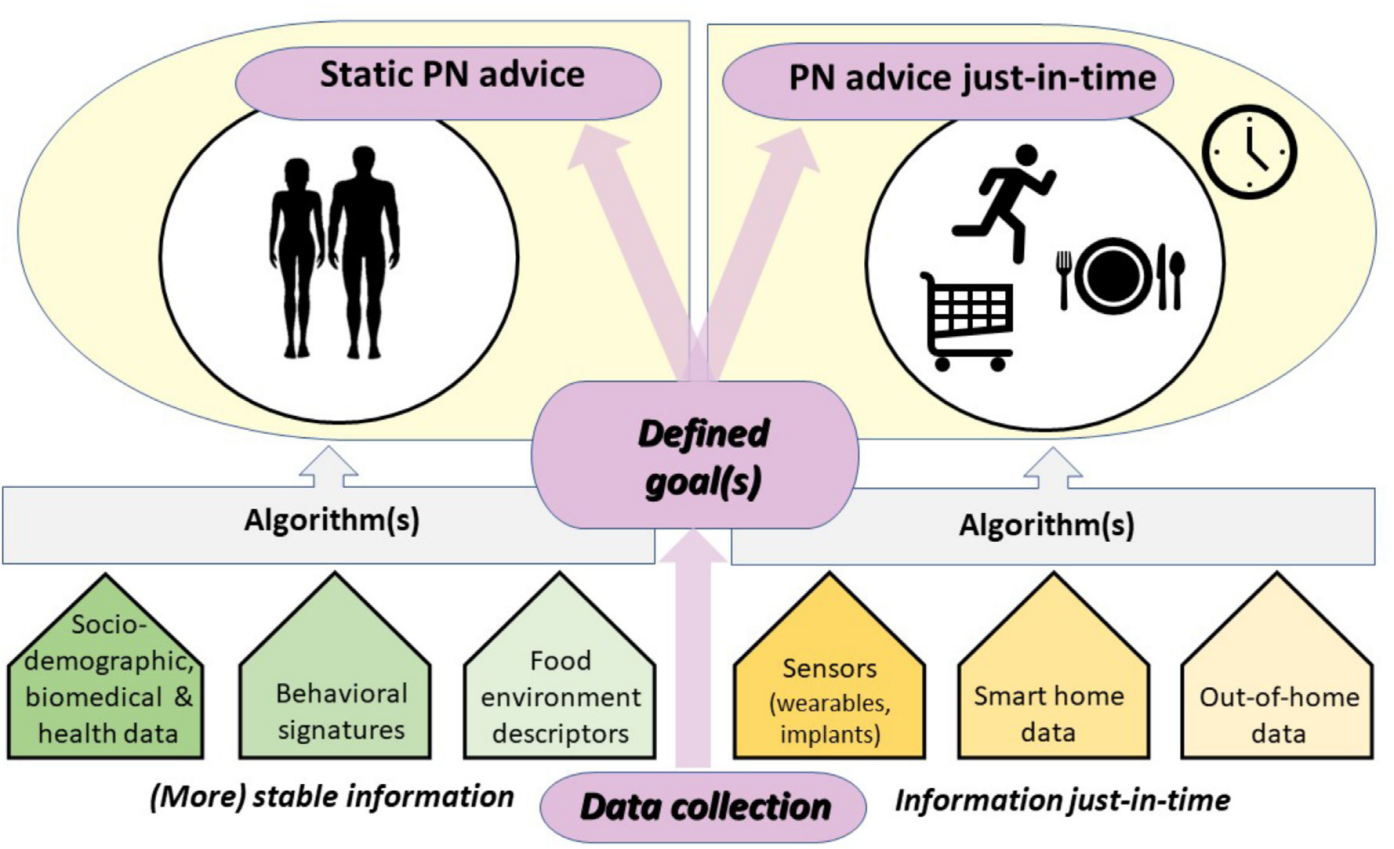
Systematic description of each person by stable and dynamic information to derive personalized nutrition (PN) advice, ideally combining general (static) advice with guidance at the moment of decision-making (*A*PNASs). *A*PNAS, Adaptive Personalized Nutrition Advice Systems.

A key feature of the *A*PNAS approach is its focus on delivering advice and services “just-in-time” at the moment of decision-making [11]. This approach aligns with evidence from other domains of behavioral change, demonstrating that timely, context-specific interventions can significantly improve outcomes. For example, JITAIs have been shown to enhance smoking cessation efforts by providing personalized prompts or coping strategies precisely when cravings are most likely to occur [79]. This just-in-time PN approach contrasts with the traditional PN model, which represents a more static concept that delivers dietary advice on a medium- to long-term basis (Figure 3). Both the *A*PNAS and conventional PN models can be applied independently or integrated, depending on the context.

As PN evolves from relying on basic, static data to adopting *A*PNASs, it necessitates specific descriptors to capture individual behavioral signatures, preferences, goals, constraints, capacities, and the surrounding food environment. Consumer smartphones, sensors, and smart-home devices, leveraging AI technologies and comprehensive databases, play a pivotal role in the success of this method. Specially, the refinement and emergence of noninvasive wearable sensors (e.g., wristwatches, tattoo-like devices, textiles, glasses, jewelry; see [80]) are increasingly enabling the multimodal, high-resolution, and even continuous real-time assessment of physical, behavioral, and biochemical parameters. The use of conservational chatbots, powered by large language models (LLMs) to deliver personalized advice, is also anticipated as part of this evolving framework.

The requirement for extended biological phenotype information in PN may be less critical depending on its focus, whether it be weight loss or choosing sustainable foods. It seems wise to leverage emerging PN systems in the digital world where consumers are actively engaged. Integrating services throughout the entire behavioral process allows for just-in-time and in-situ assistance during decision-making in real-life environments [11]. The complexity and density of input variables in digital ecosystems are set to increase significantly, driven by the delivery of real-time data on food consumption and overall lifestyle. Fitness trackers have already become seamlessly integrated into smartwatches and other devices, having demonstrated their reliability in monitoring health-related metrics. A notable advancement is the development of glucose sensors that continually report interstitial glucose profiles, offering a more comprehensive view of metabolic health [81]. It is important to note that continuous glucose monitoring is not intended as a universal recommendation but is better suited for specific contexts where detailed metabolic feedback is necessary. Recognized for their robustness and dependability, these sensors provide valuable feedback on the impact of food and drink intake on blood glucose concentrations. The visualization of metabolic responses not only delivers insightful feedback but also has the potential to significantly influence behavior and alter food choices.

**FIGURE 4:**
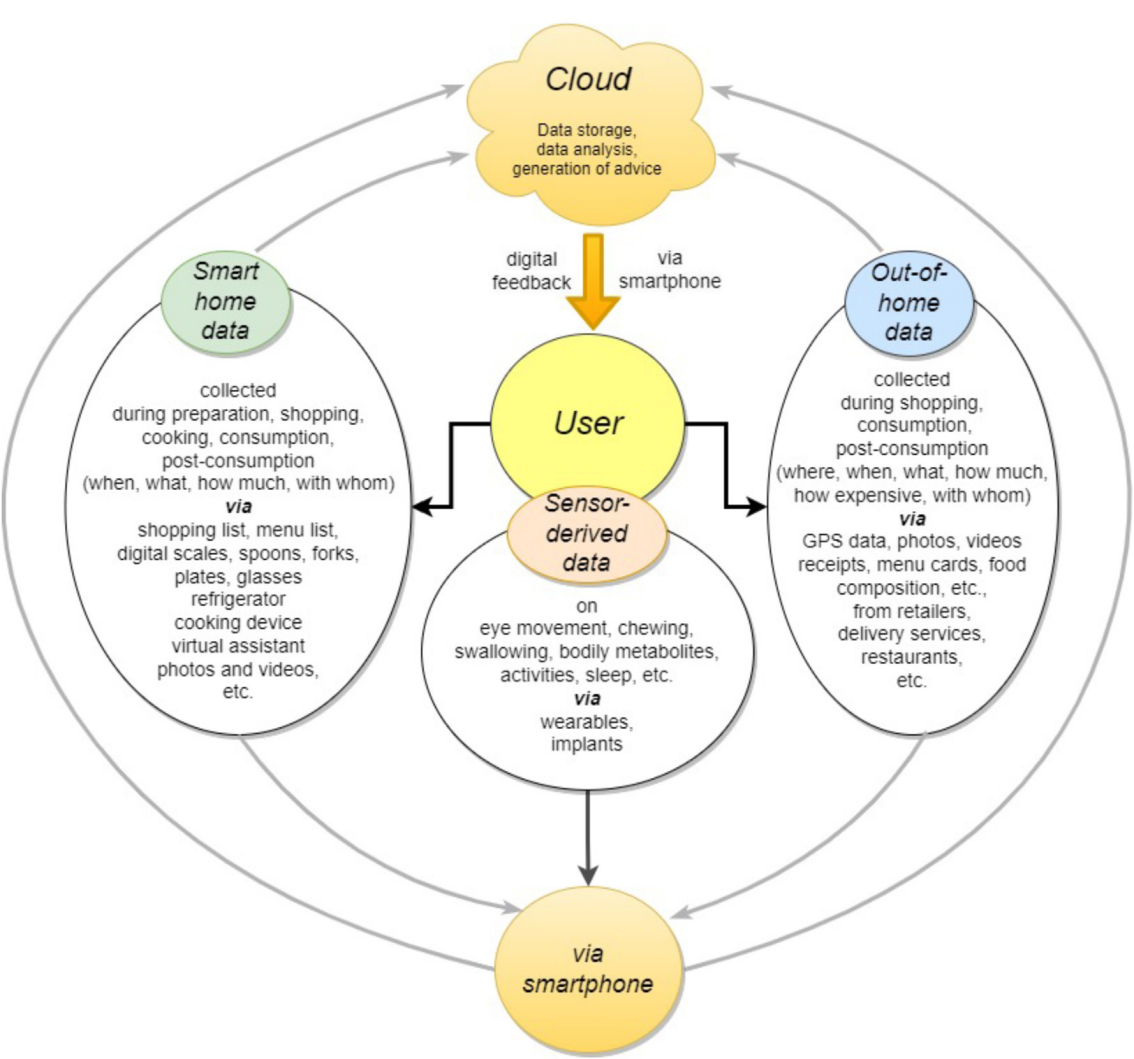
Major data sources of for PN guidance just-in-time (*A*PNAS) from the digital environment: Data from the person’s sensors, smart-home devices, and out-of-home services and activities. *A*PNAS, Adaptive Personalized Nutrition Advice Systems; PN, personalized nutrition.

Moreover, digital environments offer a multitude of innovative means for assessing behavioral signatures and dietary behavior in-situ and in time (Figure 4). For example, GPS-tracked locations of canteens, restaurants, or pick-up sites, alongside deposited menu plans (and known recipes), offer detailed insights into individuals’ meal choices and time spent at these sites [82]. In addition, it enables gathering data on social contexts (e.g., dining companions), time allocation, and financial investment. Other sources of input include shopping records for food items or foods delivered, complete with background recipes and nutrient composition. Moreover, methods such as computer vision for extracting details about food items, quantity, and composition (the latter based on a database) from images contribute to a thorough evaluation of consumed quantity and possibly an estimation of nutrient intake [50,52,53]. A more futuristic notion involves the potential integration of kitchen robots, which could take on meal preparation with pre-established recipes, facilitating in-house recording of consumption patterns [83]. Identifying the most crucial leverage points for changeable behavioral acts is essential in the implementation of PN.

These newly evolving digital ecosystems facilitate the seamless collection of an abundance of data, including dietary information and individual health parameters. Such data, captured at varying frequencies or continuously, are integrated with temporal and spatial information. The digital environment also opens novel avenues for communication and intervention, offering timely and immediate support whenever individuals need to make decisions concerning their diet, food choices, and health practices. Importantly, although data collection is crucial, seamless integration and effective use pose distinct challenges. This includes processing diverse data streams into unified systems using advanced analytics, as well as addressing ethical and legal aspects like permission, ownership, and consent. Overcoming these challenges is vital for transforming raw data into actionable insights for personalized support.

Behavioral science underscores the dynamic nature of dietary behaviors [11,46]. Dietary decisions often stem from a complex interplay of automatic and goal-directed processes. Notably, nudges and behavioral interventions drawn from the realm of psychology and economics offer promising tools for future PN strategies [84,85]. Interventions during grocery shopping, restaurant visits, or even home-deliveries, such as offering smaller portion sizes or healthier menu options, could potentially yield greater effectiveness than the application of advanced technology for omics-based phenotyping.

To effectively integrate this increasingly vast and complex array of data and provide dynamic, in-situ and just-in-time advice and services, it is essential to balance individual goals, preferences, constraints, and capabilities. Consequently, intelligent systems capable of recommending and selecting the optimal food or service based on multiple criteria are needed. Various models for DL-based recommender systems have been proposed. For example, FoodRecNet, a food recommender system, utilizes a deep artificial neural network leveraging a comprehensive set of user and food characteristics [86]. This includes basic data such as demographic information, cultural and religious background, health conditions, allergies, dietary preferences, and detailed information about food ingredients, cooking methods, and food images. Integrating this with *conversational* Al could lead to the development of chatbots for delivering tailored recommendations. For example, the potential of ChatGPT in providing PN recommendations has recently been discussed [87,88], highlighting its applicability in this evolving field. Recently, a chatbot was introduced that is powered by LLMs and specifically designed for PN advice [89].

To further enhance data integration in PN, prioritizing interoperability across devices and platforms is essential. Standardized communication protocols can facilitate seamless data exchange between wearables, mobile applications, and databases. Ensuring user-centric design in these systems—emphasizing intuitive interfaces and personalized insights—will promote engagement and adherence.

Given the sensitive nature of health and behavioral data, implementing specific, secure procedures is essential [90]. The entities hosting and providing data for PN services need to be trustworthy and operate according to legal standards [91]. However, ensuring data safety poses a significant challenge, particularly with regard to subject-identifying data. A client-centered dietary information system needs to be developed, designed to facilitate data import from digital systems and to promote active engagement among PN users.

## Conclusion

From a public health perspective, current PN approaches face limitations in effectively influencing dietary or lifestyle habits across a broad population. Addressing these challenges necessitates the development of novel strategies that expand beyond the traditional biomedical focus, incorporating individual preferences, capabilities, and goals to facilitate behavioral change within both physical and digital food environments. This also involves devising innovative methods to engage consumers who may not inherently express interest in or have the means to access such services or products, including populations with limited language proficiency or understanding [92]. Such personalized guidance should be accessible to all without being prohibitively expensive. Successfully implementing such an inclusive approach could significantly enhance the dietary quality of a substantial segment of the population and potentially yield substantial public health impact.

## Author contributions

The authors’ responsibilities were as follows – JL, BR: contributed to the conception of the work and drafted the manuscript and all authors: contributed to data collection, discussion and approval of the final version.

## Funding

German Nutrition Society is a nonprofit science organization which receives funding from the German Federal Ministry of Food and Agriculture. German Research Foundation (DFG), German Research Foundation under Germany’s Excellence Strategy (grant number: EXC 2117–422037984).

## Conflict of interest

BR leads the “Collective Appetite” project within the Centre for the Advanced Study of Collective Behaviour funded by the German Research Foundation (DFG), German Research Foundation under Germany’s Excellence Strategy (grant number: EXC 2117–422037984). JL received funding for the Joint Programming Initiative “A Healthy Diet for a Healthy Life” project DIMENSION (BMBF, grant number: 01EA1902B), for a project in the context of the Nutrition Competence Network ENABLE (BMBF, grant number: 01EA1807E), for the project RIDE-PPI funded by the Innovation Fund (G-BA, grant number: 01VSF18013), and for the project BVS III (Bavarian Ministry for Nutrition, Agriculture, and Forestry; Grant number: A/19/15). KG is a partner in the Food Nutrition Security-Cloud research consortium funded by the European Union’s Horizon 2020 Research and Innovation program (Grant Agreement No. 863059) and in the Third Bavarian Nutrition Survey (A/19/15) funded by the Bavarian State Ministry of Nutrition, Agriculture and Forestry (Bayerisches Staatsministerium für Ernährung, Landwirtschaft und Forsten, StMELF). Furthermore, KG leads the German arm of the Food&You study with funds from the Ecole Polytechnique Fédérale de Lausanne. CH leads the “Personalized nutrition and eHealth” (grant number: 01EA1709) research group within the enable cluster (Bundesministerium für Bildung und Forschung, BMBF). SL is the coordinator of the Competence Cluster for Nutrition and Cardiovascular Health (nutriCARD) Halle-Jena-Leipzig (grant numbers: 01EA1411A and 01EA1808A) funded by the German Federal Ministry of Education and Research (Bundesministerium für Bildung und Forschung, BMBF). SL has no conflicts of interest or funding disclosures relevant to this work. BW is currently president of the German Nutrition Society. All other authors report no conflicts of interest.
